# Editorial: Polarization of cellular immune response in the context of inflammation and cancer

**DOI:** 10.3389/fimmu.2024.1433808

**Published:** 2024-05-29

**Authors:** Elisa Wirthgen, Grazyna Domanska

**Affiliations:** ^1^ Department of Pediatrics, Rostock University Medical Center, Rostock, Germany; ^2^ Department of Immunlogy, University Medicine Greifswald, Greifswald, Germany

**Keywords:** cancer, polarization, immune response, inflammasome, chronic inflammation, macrophages

In the intricate landscape of immunology, the polarization of cellular immune response is a pivotal mechanism shaping the outcomes of inflammatory processes and cancer progression. This phenomenon, governed by a complex interplay of cellular players and molecular signals, holds profound implications for our understanding of disease pathogenesis and therapeutic interventions. Notably, the interplay between inflammation and cancer underscores the significance of immune polarization in shaping the tumor microenvironment and dictating cancer progression. Chronic inflammation, fueled by factors like infection, tissue injury, and autoimmune reactions, creates a protumor milieu characterized by the recruitment of immunosuppressive cells, including Tregs, myeloid-derived suppressor cells, and M2 macrophages. This immunosuppressive network orchestrates a state of immune evasion, shielding cancer cells from immune surveillance and facilitating tumor growth, invasion, and metastasis. Within this Research Topic, six articles discuss new aspects addressing the relevance of polarization in the context of inflammation and cancer. Moreover, new mechanistic insights into regulating inflammasomes may help identify targets for developing innovative therapeutic approaches.

In the inflamed tissue, macrophages can gain the ability to produce and secrete inflammatory mediators and exhibit pro-inflammatory and later anti-inflammatory functions. Zhao et al. investigated the role of bile acid-induced pyroptosis in macrophages in the context of gastric carcinogenesis. The authors proved that bile acids could enhance the expression and subsequent release of the protein HMGB1 due to enhancing inflammasome activation in macrophages. As a part of the innate immune system, inflammasomes are stimulus-induced cytoplasmic multimeric protein complexes. They are assumed to influence tumorigenesis by regulating innate and adaptive immune systems. According to the described tumor-promoting effects of increased HMGB1 release, the authors concluded that targeting this protein could potentially emerge as an innovative target for clinical interventions to treat gastric cancer. The role of inflammation as a key characteristic of tumor development was also discussed by (Deng et al.). They focused their review on the dual role of inflammasomes in the occurrence and development of different tumors, including tumor suppression and tumor promotion. Mouse models of carcinogenesis indicate that specific inflammasomes promote tumorigenesis by suppressing NK cells and T cell activity.

Moreover, they mediate the recruiting of immune cells with strong immunosuppressive activity, resulting in a tumor-promoting microenvironment. Hence, inflammasomes can be targeted or modulated to achieve tumor immunotherapy. Accordingly, several experimental approaches target inflammasome activity by, e.g., blocking upstream signaling pathways, inhibiting inflammasome components, or antagonizing the end products of inflammasome activation. In clinical studies, inhibition of cytokine signaling has been the most successful approach, e.g., blocking the IL1 receptor. The inflammasome activation was also part of a study by (Zhi et al.). In their study, the authors investigated the effect of the antimicrobial peptide Cecropin AD (CAD) on mucosal injury in chickens. The results indicate that CAD attenuated intestinal damage of the mucosa, experimentally induced by lipopolysaccharides. These effects were related to anti-oxidative and anti-inflammatory actions, including attenuating the expression and activation of the NLRP3 inflammasome associated with reduced production of inflammatory cytokines.

During cancer therapy, patients often have to take anti-resorptive medication such as zoledronate (Zol), which belongs to the class of drugs known as bisphosphonates. One of the most feared side effects is medication-induced osteonecrosis of the jawbone (MRONJ). Struckmeier et al. investigated the role of macrophages in promoting the development of MRONJ in an experimental model using Wistar rats. They observed a significantly higher number of macrophages and a heightened pro-inflammatory environment in the mandible compared to the tibia. Moreover, the percentage of anti-inflammatory macrophages (CD163+ M2 macrophages) decreased. The most significant decrease in anti-inflammatory macrophages was observed after the combinatorial tooth extraction treatment and the previous Zol application. A shift from M1 to M2 macrophages and restoration of a balanced M1/M2 ratio might reduce the risk of developing an MRONJ and improve treatment options. The polarization of macrophages was also reviewed by Li et al., focusing, in particular, on the role of pyruvate dehydrogenase complexes (PDCs), one of the main regulators of metabolic activity in mammals. PDC activity is primarily modulated by four isozymes of pyruvate dehydrogenase kinase (PDK), which regulate metabolic flexibility and contribute to energy homeostasis by negatively moderating PDC activity. They further modify the polarization of macrophages by regulating the metabolic transformation from OXPHOS (M2) to aerobic glycolysis (M1), which may promote inflammatory diseases such as diabetes or cardiovascular diseases. Accordingly, several small-molecule inhibitors of PDK have been identified, some of which are currently being investigated in animal models of metabolic and inflammation-related diseases. The polarization of monocyte-derived macrophages modulated by extrinsic factors was investigated by Franzoni et al., who investigated the immunomodulatory effects of milk extracellular vesicles (mEVs) of goats, which have drawn increasing interest due to their potential biomedical applications. Interestingly, the results indicate that goat mEVs polarized macrophages toward a pro-inflammatory and tumoricidal M1-like phenotype, but they did not have a significant impact in case these cells were already classically activated (moM1). The authors concluded that administering mEVs loaded with therapeutic molecules might guarantee more efficient delivery of these molecules and contribute to reprogramming tumor-associated macrophages toward an M1-like tumoricidal phenotype.

In conclusion, the results of this Research Topic underline the significance of cellular polarization regarding the outcomes of inflammatory processes and cancer progression. The focus was on mediators of the innate immune response. We anticipate these studies will provide state-of-the-art information about mechanisms underlying immune polarization and open additional areas for future investigation on disease pathogenesis and therapeutic interventions in the context of precision immunotherapy.

## Author contributions

EW: Writing – review & editing, Writing – original draft. GD: Writing – review & editing.

